# Predicting radiation pneumonitis in lung cancer: a EUD-based machine learning approach for volumetric modulated arc therapy patients

**DOI:** 10.3389/fonc.2024.1343170

**Published:** 2024-01-31

**Authors:** Fengsong Ye, Lixia Xu, Yao Ren, Bing Xia, Xueqin Chen, Shenlin Ma, Qinghua Deng, Xiadong Li

**Affiliations:** ^1^ Department of Tumor Radiotherapy and Chemotherapy, Lishui People’s Hospital, Lishui, China; ^2^ Medical Imaging and Translational Medicine Laboratory, Hangzhou Cancer Center, Hangzhou, China; ^3^ Department of Radiation Oncology, Hangzhou Cancer Hospital, Zhejiang, Hangzhou, China

**Keywords:** lung equivalent uniform dose, radiation pneumonitis, machine learning, normal tissue complication probability, prediction

## Abstract

**Purpose:**

This study aims to develop an optimal machine learning model that uses lung equivalent uniform dose (lung EUD to predict radiation pneumonitis (RP) occurrence in lung cancer patients treated with volumetric modulated arc therapy (VMAT).

**Methods:**

We analyzed a cohort of 77 patients diagnosed with locally advanced squamous cell lung cancer (LASCLC) receiving concurrent chemoradiotherapy with VMAT. Patients were categorized based on the onset of grade II or higher radiation pneumonitis (RP 2+). Dose volume histogram data, extracted from the treatment planning system, were used to compute the lung EUD values for both groups using a specialized numerical analysis code. We identified the parameter α, representing the most significant relative difference in lung EUD between the two groups. The predictive potential of variables for RP2+, including physical dose metrics, lung EUD, normal tissue complication probability (NTCP) from the Lyman-Kutcher-Burman (LKB) model, and lung EUD-calibrated NTCP for affected and whole lung, underwent both univariate and multivariate analyses. Relevant variables were then employed as inputs for machine learning models: multiple logistic regression (MLR), support vector machine (SVM), decision tree (DT), and K-nearest neighbor (KNN). Each model's performance was gauged using the area under the curve (AUC), determining the best-performing model.

**Results:**

The optimal α-value for lung EUD was 0.3, maximizing the relative lung EUD difference between the RP 2+ and non-RP 2+ groups. A strong correlation coefficient of 0.929 (P< 0.01) was observed between lung EUD (α = 0.3) and physical dose metrics. When examining predictive capabilities, lung EUD-based NTCP for the affected lung (AUC: 0.862) and whole lung (AUC: 0.815) surpassed LKB-based NTCP for the respective lungs. The decision tree (DT) model using lung EUD-based predictors emerged as the superior model, achieving an AUC of 0.98 in both training and validation datasets.

**Discussions:**

The likelihood of developing RP 2+ has shown a significant correlation with the advancements in RT technology. From traditional 3-D conformal RT, lung cancer treatment methodologies have transitioned to sophisticated techniques like static IMRT. Accurately deriving such a dose–effect relationship through NTCP modeling of RP incidence is statistically challenging due to the increased number of degrees-of-freedom. To the best of our knowledge, many studies have not clarified the rationale behind setting the α-value to 0.99 or 1, despite the closely aligned calculated lung EUD and lung mean dose MLD. Perfect independence among variables is rarely achievable in real-world scenarios. Four prominent machine learning algorithms were used to devise our prediction models. The inclusion of lung EUD-based factors substantially enhanced their predictive performance for RP 2+. Our results advocate for the decision tree model with lung EUD-based predictors as the optimal prediction tool for VMAT-treated lung cancer patients. Which could replace conventional dosimetric parameters, potentially simplifying complex neural network structures in prediction models.

## 1 Introduction

Radiation pneumonitis (RP) is a critical complication for lung cancer patients undergoing radiation therapy (RT). The incidence of acute RP ranges between 5% and 36%, with respiratory failure subsequent to RP being a leading cause of death in these patients (1). The emergence of RP not only challenges treatment effectiveness but also significantly diminishes patients’ quality of life (1). As such, accurately predicting RP and initiating timely interventions are crucial to prevent severe radiation-induced lung injury.

The prevailing method to predict radiation-induced pneumonitis in clinical practice adopts the dose-volume threshold approach. This method analyzes the volume of lung tissue receiving a specific radiation dose, termed V_dose, using techniques like conformal 3D-CRT or intensity modulated radiotherapy (IMRT). While many studies (2, 3) have reported a correlation between V_dose and RP occurrence (4), others (5, 6) found challenges such as incomplete data or nonlinear patterns, hindering the establishment of a solid correlation threshold. With the advent of advanced radiation techniques like volumetric modulated arc therapy (VMAT) and tomotherapy (TOMO), there’s been an upsurge in the lung’s low-dose volume. For instance, Shen WB et al. underscored V_5 (volume of lung receiving a low radiation dose) as a key predictor for grade 2 or higher radiation pneumonitis (RP 2+) in patients receiving radiotherapy for middle and lower thoracic esophageal cancer (7). Presently, RP prediction mainly hinges on specific dose volume thresholds derived from lung dose volume histograms (DVH), potentially missing essential data pertinent to RP development.

In this study, we undertook the formulation and comparative assessment of diverse machine learning models designed to predict RP 2+ under non-uniform radiation conditions. We centered our investigation on the lung equivalent uniform dose (lung EUD) and its parameter α, both exhibiting strong correlation with RP 2+ prediction. Our utilized machine learning algorithms encompass decision trees (DT), K-nearest neighbor (KNN), logistic regression (LR), and support vector machine (SVM).

Niemerko et al. initially introduced the concept of equivalent uniform dose (EUD) grounded in the “Weber-Fechner-Stevens” law, suggesting that two distinct dose distributions are equivalent if they induce identical radiobiological effects (8). Consequently, EUD can be understood as the dose of uniform radiation inducing a radiobiological effect akin to non-uniform radiation (9). Within this context, the α parameter in EUD is pivotal, signifying the tissue’s radiation dose tolerance; higher α values denote increased tolerance. Different α values categorize diverse tissue types, adhering to the “Stevens law”. Tumor tissue exhibits a negative α value, suggesting that under-dosing promotes unchecked tumor growth. In contrast, serial organs in normal tissue possess high α values due to heightened vulnerability to large doses, while parallel organs have diminished α values reflecting their lesser dose sensitivity.

The EUD formula is conceptualized through a stimulus-response model, as illustrated in Equation 1. For streamlining the correlation analysis between the risk of RP 2+ and lung irradiation, we judiciously chose a pertinent α value. This choice predicates on viewing RP 2+ as a biological consequence of external radiation therapy. We also furnish the optimal α value, a significant lung tissue descriptor, facilitating the precise prediction of RP 2+.

## 2 Materials and methods

### 2.1 Patients

We meticulously assembled a retrospective cohort of 77 patients diagnosed with locally advanced squamous cell lung cancer (LASCLC) from July 2017 to February 2018. Each patient, as part of their concurrent chemoradiotherapy, received weekly nab-paclitaxel doses of 60 mg/m^2 and carboplatin with an area under the plasma concentration-time curve (AUC) of 2. They underwent a thoracic radiation therapy regimen of 66 Gy over 33 fractions. Post radiation, consolidation chemotherapy, entailing nab-paclitaxel (260 mg/m^2 on day 1) and carboplatin (AUC 6 on day 1), was administered. The cohort included 42 patients on a weekly regimen—22 with paclitaxel, 6 with cisplatin, 5 with pemetrexed plus cisplatin, and 9 with paclitaxel plus carboplatin. The remaining 35 patients followed a 4-week regimen—16 with docetaxel plus cisplatin, and 19 with pemetrexed plus carboplatin. **Table 1** provides a concise overview of the patients’ clinical attributes.

**Table 1:** Clinical characteristics of the patients (n=77).

Characteristics	Without RP 2+ (percent)	With RP 2+ (percent)	p values
Patients	55 (71.4%)	22 (28.6%)	0.35
Sex			0.24
Male	58(75.3%)	16(72.7%)	
Female	19(24.7%)	6(27.3%)	
Median age, y (IQR)	67(12)	71(11)	0.45
Charlson rate			0.21
0-2	72(93.5%)	5(6.5%)	
3-4	4(5.2%)	73(94.8%)	
5-6	1(1.3%)	76(98.7%)	
Tumor location			0.22
LLL	13(16.9%)	4(18.2%)	
LUL	14(18.2%)	2(9.1%)	
RLL	39(50.6%)	13(59.1%)	
RUL	11(14.3%)	3(13.6%)	
Smoking history			0.57
Ever	41(53.2%)	7(31.8%)	
Never	36(46.8%)	15(68.2%)	
Radioprotectant			0.76
With usage	26(33.8%)	8(36.4%)	
Without usage	51(66.2%)	14(63.6%)	

LLL, left lower lobe; LUL, left upper lobe; RLL, right lower lobe; RUL, right upper lobe.

### 2.2 Radiation therapy techniques

**Figure 1:**
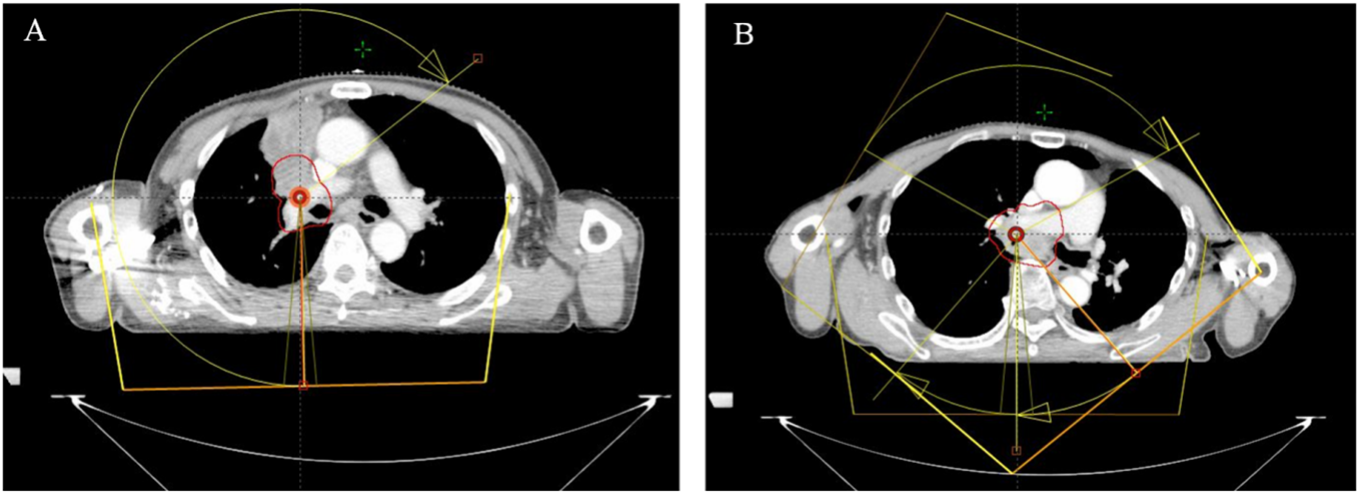
Illustration of VMAT treatment arcs for tumors located in one side of the lung **(A)** and in the central mediastinum of the lung **(B)**. (Treatment arcs are shown in yellow with arrows, PTV is displayed with the red solid line.).

Patients were positioned using thermoplastic films and underwent CT simulation scans with the Philips Brilliance Big Bore CT scanner covering from the upper margin of the liver to the cricoid cartilage. Dose distributions, calculated with the Pinnacle (V9.8) treatment planning system, were delivered via Volumetric Modulated Arc Therapy (VMAT). Treatment volumes were delineated as per the Radiation Therapy Oncology Group (RTOG) atlas. The planning target volume (PTV) was derived from the clinical target volume (CTV) with specific expansions. For tumors in one lung, a single arc VMAT minimized impact on healthy lung tissue (**Figure 1A**). For central mediastinum tumors, a 3-arc VMAT approach was chosen, resembling static intensity-modulated radiation therapy (IMRT) (**Figure 1B**). Prior to each session, isocentric validation was executed using cone-beam CT (CBCT) and any needed repositioning was conducted with the HexaPOD six-dimensional couch adjustment.

### 2.3 Evaluation criterion for radiation pneumonitis

Post radiation therapy (RT), patients were evaluated within six months to confirm the diagnosis and grade of radiation pneumonitis, the patient’s clinical symptoms, imaging studies, and laboratory test results are reviewed and evaluated by a clinical team or a radiologist using the Radiation Therapy Oncology Group (RTOG (10)) classification criteria for acute radiation lung injury. Those manifesting RP 2+(Grade2: Persistent cough requiring narcotic, antitussive agents or dyspnea with minimal effort but not at rest; Grade3: Severe cough unresponsive to narcotic antitussive agent or dyspnea at rest/clinical or radiological evidence of acute pneumonitis/intermittent oxygen or steroids may be required; Grade4: Severe respiratory insufficiency/continuous oxygen or assisted ventilation.) were categorized as the radiation pneumonitis group (GRP), and others as the non-radiation pneumonitis group (G-NRP).

### 2.4 Lung EUD calculation

Data, including DVH and lung volume metrics, were extracted from the Pinnacle treatment planning system. Each voxel’s radiation dose (D_i) in the lung was computed using MATLAB software (version R2011b). lung EUD values for the bilateral and unilaterally affected lung were calculated employing Equation 1 from Zhou et al. (11), where α, an organ EUD characteristic parameter, was constrained between -50 and +50 in the Pinnacle system due to limitations. The relative lung EUD difference between GRP and G-NRP groups was determined using Equation 2. By exploring the α-R correlation, we identified the specific α value that best differentiated average lung EUD values between the groups. This aided in understanding the relationship between this determined lung EUD value and traditional volume dose thresholds.


(1) EUD=(1N∑Diα)1α,

(2) $$\text{R}=\frac{\left[\text{lung EUD}\left(\text{GRP}\right)-\text{lung EUD}\left(\text{G}-\text{NRP}\right)\right]}{\text{lung EUD}\left(\text{G}-\text{NRP}\right)}\times 100\%.$$

### 2.5 NTCP calculation

The NTCP_LKB and lung EUD calibrated NTCP_lung EUD for each patient were determined using the Lyman-Kutcher-Burman (LKB) model, as described by Equations 3, 4. In this study, all NTCP calculations were specific to the affected and entire lung.


(3) $$\begin{array}{l} NTCP=\frac{1}{\sqrt{2\pi }}\int_{-\infty }^{t}e^{-\frac{t^{2}}{2}}dt\\ t=\frac{d_{ref}-TD_{50}(v_{eff})}{m\cdot TD_{50}(v_{eff})}\\ v_{eff}=\sum_{i}v_{i}\cdot {\left(d_{i}/d_{ref}\right)}^{1/n} \end{array}$$

(4) $$NTCP\_LEUD=\frac{1}{1+{\left(\frac{TD_{50}}{LEUD}\right)}^{4\gamma 50}}$$

The model contains four parameters: TD50, slope parameter γ50 or m, and a parameter n to account for the strength of the volume dependence of the tolerance dose as a power law. TD50 is the dose to the reference volume (usually the whole organ), for which there is a 50% probability of complications occurring. The parameter γ50 or m defines the slope of the dose-complication probability curve. Veff was set to either the whole organ (liver data) or the largest irradiated volume (spinal cord data) for this study. Where vi is each irradiated fractional sub volume (i = 1,……, n, ∑i vi =1) irradiated with dose di and the reference dose dref. The specific values for these parameters are as follows: TD50 = 24.5 Gy, γ50 = 2, dref=2, and m=2.

### 2.6 Statistical analysis

Performed with R version 4.1.1, statistical analysis incorporated the independent sample t-test to probe variables and correlation analysis to study relationships between Vdose, lung EUD (optimal), and RP 2+ occurrence. A significance level of P< 0.05 was used to discern any significant difference in RP 2+ incidence between GRP and G-NRP. The Area Under the Curve (AUC) in the Receiver Operating Characteristic (ROC) analysis gauged the predictive efficacy of potential predictors.

### 2.7 Machine learning methods

Post multivariate regression analysis and AUC computation, significant variables underwent training and validation of machine learning models. Four algorithms—multiple logistic regression (MLR), SVM, DT, and KNN—were utilized. We allocated 70% of the dataset for training and the remainder for validation, with model building incorporating a 5-fold cross-validation. Models were crafted using the Sklearn package in Python (Version 3.8.10). The AUC of the ROC curve assessed each model’s predictive performance.

## 3 Results

### 3.1 Incidence of RP 2+

Based on the adverse reaction assessment criteria set by the Radiation Therapy Oncology Group (RTOG) in the U.S., we began evaluating acute reactions from the start of radiotherapy. During the monitoring period, 22 patients, or 28.6% of the study group, exhibited RP 2+ symptoms. Conversely, 55 patients did not show any discernible RP signs.

### 3.2 Variation in lung EUD value among GRP and G-NRP for different α values

**Figure 2:**
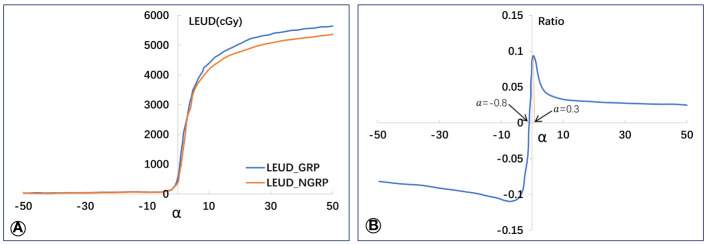
Average values of lung EUD in GRP and G-NPR groups **(A)** and the relative lung EUD difference **(B)** between the two groups.

**Figure 2A** depicts the rising trend of lung EUD values for both GRP and G-NRP with increasing α value. Notably, at the lower dose range, lung EUD (G-NRP) surpasses lung EUD (GRP) until α approaches -0.8 (**Figure 2B**). Beyond this threshold, the lung EUD (G-NRP) declines below lung EUD (GRP) for α values greater than -0.8. The most pronounced relative difference in lung EUD values between the groups with and without radiation pneumonitis peaks at α = 0.3.

### 3.3 Physical and biological dosimetric metrics between GRP and G-NRP

An independent sample t-test was employed to explore the link between the risk of RP 2+ and pertinent factors, such as planning target volume (Vol_PTV), bilateral lung volume (Vol_Lung), PTV-to-lung ratio, and more. **Table 2** provides a concise summary. Significant differences in parameters like V5, V10, V20, V30, MLD, and lung EUD for the lung were observed between groups (with P-values stated). However, no significant differences in terms of Vol_Lung, Vol_PTV, and others were detected as their P-values were above 0.05.

**Table 2:** T test for Dose-related parameters between GRP and G-NRP.

	GRP			G-NRP				
	N	Average	SD	N	Average	SD	t	P
Vol_Lung	22	3155.60	925.76	55	3085.38	873.63	-0.332	0.741
Vol_PTV	22	474.77	276.33	55	391.15	248.15	-1.347	0.183
PTV-to-lung ratio	22	0.16	0.11	55	0.13	0.081	-1.360	0.180
V5	22	44.07	6.24	55	39.55	9.55	-2.292	0.025
V10	22	32.60	5.26	55	28.17	7.59	-2.794	0.007
V20	22	22.73	3.66	55	19.70	6.51	-2.327	0.023
V30	22	15.87	3.27	55	13.55	5.24	-2.162	0.034
MLD	22	1239.87	210.19	55	1094.13	301.65	-2.310	0.024
lung EUD(= 0.3)	22	627.94	187.57	55	510.23	165.69	-2.887	0.005
NTCP_LKB_Sick	22	0.083	0.038	55	0.039	0.024	6.172	0.000
NTCP_LKB_Total	22	0.076	0.023	55	0.054	0.027	3.800	0.000
NTCP_lung EUD_Sick	22	0.195	0.065	55	0.047	0.209	10.235	0.000
NTCP_lung EUD_Total	22	0.116	0.053	55	0.032	0.019	10.318	0.000
SAA	22	377.80	102.84	55	384.13	89.34	0.277	0.783

MLD, mean lung dose; SAA, sum of the arc angle; NTCP_LKB_Sick, NTCP_LKB for the affected lung; NTCP_LKB_Total, NTCP_LKB for the entire lung.

As shown in **Table 2**, significant differences were observed in several key parameters when comparing the groups with and without RP 2+. These differences were particularly notable in V5, V10, V20, V30, MLD, and lung EUD for the lung, with statistically significant values (P = 0.025, P = 0.007, P = 0.023, P = 0.034, P = 0.024, P = 0.005, respectively). Additionally, there were significant disparities in the NTCP calculations for both the affected lung and the entire lung between the two groups (P = 0.000). However, no statistically significant distinctions were observed between the groups in terms of Vol_Lung, Vol_PTV, PTV-to-Lung ratio, and SAA, as the corresponding P-values exceeded 0.05.

### 3.4 Correlation between traditional dosimetric metrics and lung EUD

**Table 3**’s correlation analysis reveals that when α is 0.3, the calculated lung EUD value strongly correlates with several dosimetric metrics. However, as α changes, the correlations with traditional metrics decline, with some exceptions.

**Table 3:** Correlation between dosimetric metrics and lung EUD calculated at different α values.

		V5	V10	V20	V30	MLD
lung EUD(α=0.3)	person p	0.936 0.000	0.911 0.000	0.914 0.000	0.923 0.000	0.961 0.000
lung EUD(α=1)	person p	0.374 0.002	0.424 0.000	0.444 0.000	0.606 0.000	0.868 0.000

### 3.5 Multivariate analysis of RP 2+ based on lung EUD and dosimetric metrics

A multivariate regression analysis identified factors linked to the risk of RP 2+. Only factors with P< 0.05 were considered significant. Due to the robust correlation among specific lung metrics, they were amalgamated into a single term in the analysis. As evidenced in **Table 4**, several factors were identified as independent predictors of RP 2+.

**Table 4:** Multivariate regression analysis for prediction of RP 2+.

	B	S.E,	Wals	P-value
Physical dose*	0.002	0.000	4.340	0.027
lung EUD(α = 0.3)	0.004	0.002	5.576	0.018
PTV-to-lung ratio	0.140	3.427	0.002	0.967
NTCP_LKB_SICK	0.002	0.001	-3.091	0.001
NTCP_lung EUD_SICK	0.917	0.306	2.990	0.002
NTCP_lung EUD_TOTAL	0.409	0.243	0.168	0.886
NTCP_LKB_TOTAL	0.129	0.069	1.861	0.063
Constant	-3.205	1.088	8.671	0.003

Physical dose*= MLD *V5 * V10 * V20 * V30.

### 3.6 ROC analysis of predictors

**Figure 3:**
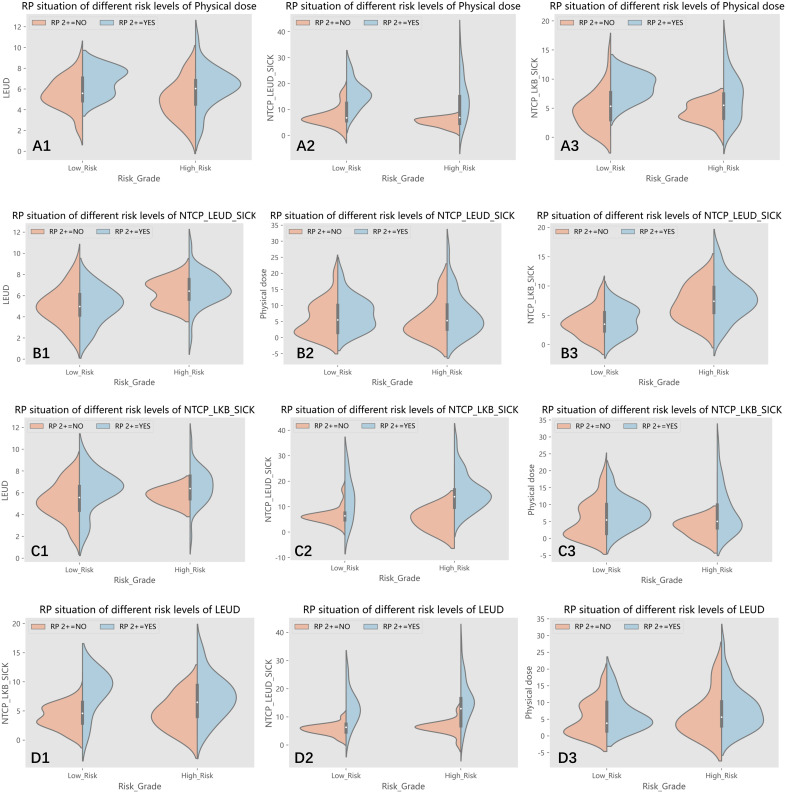
(A1-A3) The patients were divided into low-risk and high-risk groups according to the cut off value of physical dose, and the distribution of lung EUD, NTCP_lung EUD_SICK and NTCP_LKB_SICK values in two groups are shown, respectively; (B1-B3) The patients were divided into low-risk and high-risk groups according to the cut off value of NTCP_lung EUD_SICK, and the distribution of lung EUD, physical dose and NTCP_LKB_SICK values in two groups are shown, respectively; (C1-C3) The patients were divided into low-risk and high-risk groups according to the cut off value of NTCP_LKB_SICK, and the distribution of lung EUD, NTCP_lung EUD_SICK and physical dose values in two groups are shown, respectively; (D1-D3) The patients were divided into low-risk and high-risk groups according to the cut off value of lung EUD, and the distribution of NTCP_LKB_SICK, NTCP_lung EUD_SICK and physical dose values in two groups are shown, respectively.

The Receiver Operating Characteristic (ROC) curve analysis assessed the efficacy of predictive factors for RP 2+. **Table 5**’s comprehensive results spotlight the predictive potential of specific parameters. Furthermore, the data was segmented into low-risk and high-risk groups based on variable cutoffs. **Figure 3**’s violin plots elucidate the variable distributions within these groups, revealing the challenges in pinpointing RP2+ based solely on physical dose cutoffs.

**Table 5:** AUC calculation of predictors for prediction of RP 2+.

Parameter	AUC	cut-off value	95% CI
Physical dose*	0.643	6.075	4.342~9.324
lung EUD(α = 0.3)*	0.695	598 cGy	0.572~0.818
PTV-to-lung ratio	0.434	0.156	0.078~0.234
NTCP_LKB_SICK*	0.826	6.55%	0.725~0.927
NTCP_lung EUD_SICK*	0.862	7.72%	0.900~1.000
NTCP_lung EUD_TOTAL	0.815	5.51%	0.824~1.000
NTCP_LKB_TOTAL	0.737	5.55%	0.627~0.847

Physical dose*= MLD *V5 * V10 * V20 * V30.

Variables that exhibited a significance level below 0.05 in the multivariate regression analysis were further investigated. The dataset was subsequently divided into two categories: a low-risk group and a high-risk group, determined by the respective cutoff values of each selected variable. These divisions enabled the generation of violin plots, depicted in **Figure 3**, illustrating the distribution of the remaining variables within both groups: those with RP2+ and those without it. To provide an example, the dataset was split into two subsets based on the 6.075 Gy cutoff for physical dose. The violin plots visually represented the distributions of other selected variables, namely lung EUD, NTCP_LKB_SICK, and NTCP_lung EUD_SICK, in both groups with and without RP2+.

As depicted in **Figure 3**(A1), an interesting observation emerges when categorizing the groups based on the physical dose cutoff. Some samples in the low-risk group exhibited RP2+, while a few samples in the high-risk group did not show RP2+. It becomes apparent that patients with RP2+ in the low-risk group had lung EUD values clustering around 8. Conversely, those in the high-risk group without RP2+ had predominantly lower lung EUD values, mainly below 6. This observation highlights the challenge of accurately identifying RP2+ solely based on the physical dose cutoff. The relatively low AUC value of 0.643 associated with physical dose in predicting RP2+ further validates this predicament.

Upon closer examination of the high-risk group in B1) and the low-risk group in B2, as illustrated in **Figure 3**, an intriguing pattern emerges in the samples of RP2+-negative patients. These samples exhibit a phenomenon similar to Gaussian mixed distribution. This observation prompts the realization that relying solely on the cutoff value of NTCP_lung EUD_SICK may not be sufficient for precise classification of patients. Importantly, this multifaceted distribution pattern is also evident in figures D1 and D3 using lung EUD as the classification parameter, as well as in figure C3 utilizing NTCP_LKB_SICK as the classification parameter. Taken together, these findings suggest that our sample dataset cannot be linearly separated, underscoring the need for a multi-parameter, nonlinear machine learning algorithm to achieve more accurate classification and prediction.

### 3.7 Machine learning models’ predictive performance

**Figure 4:**
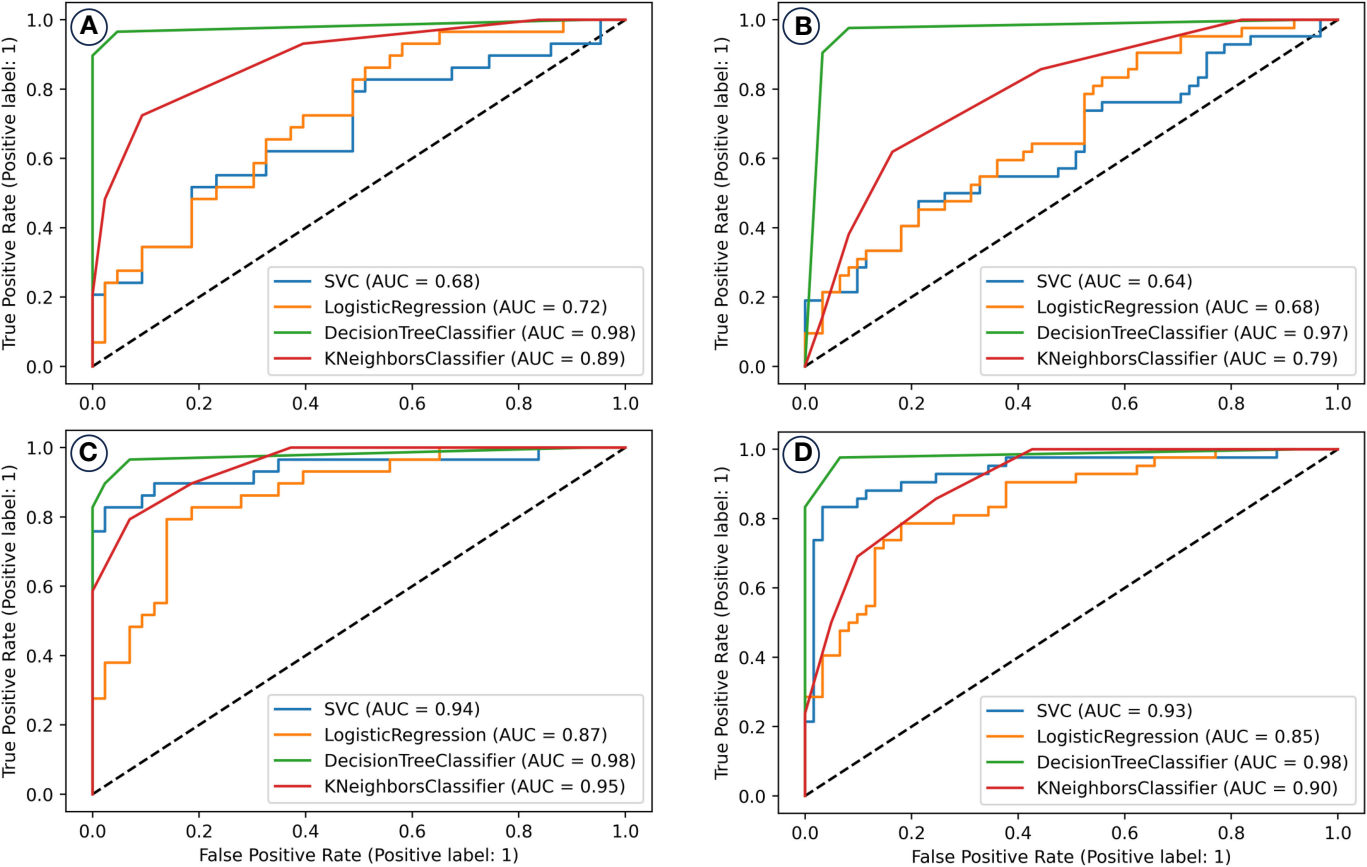
The receiver operating characteristic (ROC) curves of machine learning models for the prediction/classification of patients with and without radiation pneumonitis of grade two or higher (RP 2+). Prediction model performance in the training set **(A)** and validation set **(B)** without including the lung EUD and NTCP_lung EUD_SICK in the model building; Prediction model performance in the training set **(C)** and validation set **(D)** with including the lung EUD and NTCP_lung EUD_SICK in the model building. AUC, area under the curve; SVM, support vector machine.

Selected variables were used as inputs for machine learning models. Two distinct predictor sets were formed and subjected to model training. **Figure 4** showcases the receiver operating characteristic (ROC) curves of various models, indicating the superiority of models integrating lung EUD-based predictors over those that don’t. Specifically, the SVM model with lung EUD predictors markedly excelled.

The Decision Tree (DT) model, enriched with lung EUD predictors, was the standout, achieving an AUC of 0.98 in both training and validation datasets.

**Figure 5:**
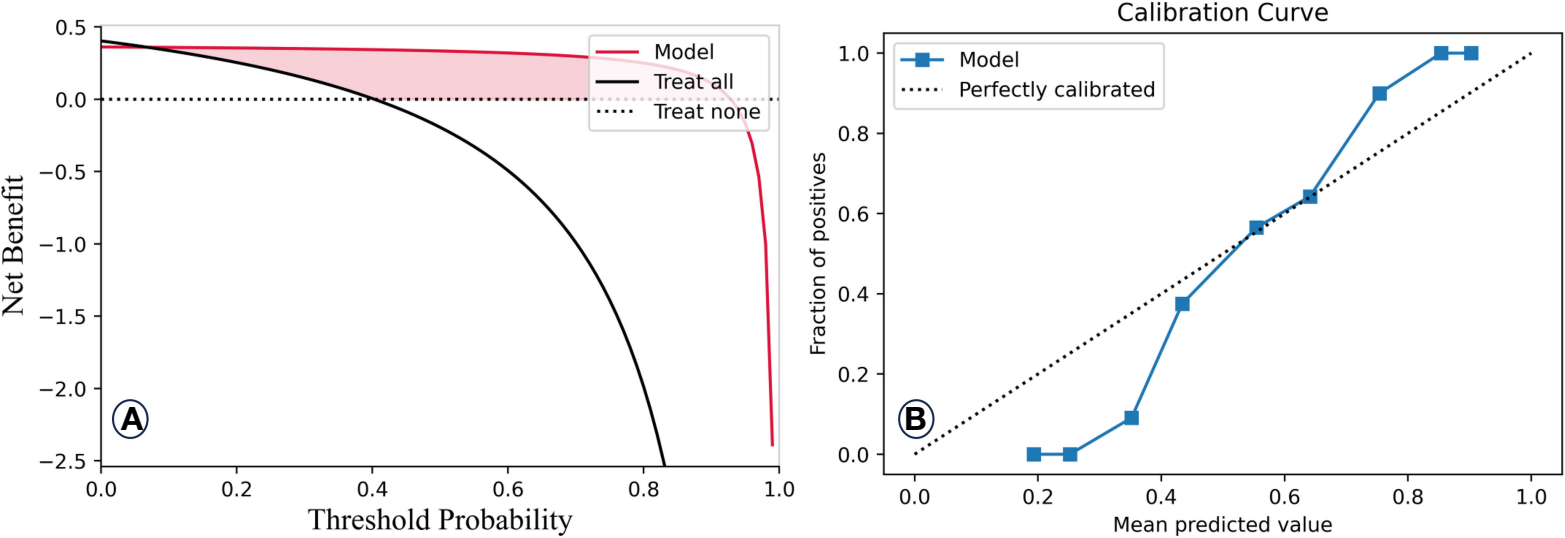
DAC curve **(A)** and model calibration curve **(B)** of the final decision tree mode.

Lastly, the final DT model’s exhaustive evaluation is displayed in **Figure 5**, highlighting its commendable prediction benefits and calibration. This model’s predictions closely align with patients who can benefit clinically, with a calibration curve mirroring an ideal curve. This alignment emphasizes the model’s credibility in predicting RP2+ probabilities.

Ultimately, following a 5-fold cross-validation process in both the training and validation datasets, we identified the two types of models that exhibited the most exemplary performance. These outcomes are visually represented in **Figure 4**.

Evidently, the models constructed with the inclusion of lung EUD-based predictors demonstrate superior prediction performance in contrast to those constructed without such predictors. Notably, the SVM model, which incorporates lung EUD-based predictors, significantly outperforms its counterpart without them. For instance, in the training set, the SVM model inclusive of lung EUD-based predictors attains an impressive AUC of 0.94, as opposed to 0.68 in the model lacking these predictors.

Of all the models considered, the DT model, when enriched with lung EUD-based predictors, emerges as the top performer, boasting an AUC value of 0.98 in both the training and validation sets, thereby showcasing its exceptional predictive capabilities. We conducted a comprehensive evaluation of the final DT classification model by assessing its net benefits and calibration outcomes, as illustrated in **Figure 5**. In **Figure 5A**, discernible patterns emerge, wherein patients who stand to derive clinical benefits from the model’s predictions are concentrated within the red area. In alignment with the tenets of the DAC algorithm, this model approximates an ideal model. Notably, upon implementing interventions guided by our DT model’s predictions, the net benefits for patients exhibit only marginal reductions as the threshold value increases. Furthermore, the calibration analysis of the model reveals that its calibration curve closely aligns with the distribution of an ideal calibration curve. This congruence underscores that the model’s predicted probabilities for RP2+ closely mirror the empirical probabilities, thereby enhancing its reliability.

## 4 Discussion

The likelihood of developing RP 2+ has shown a significant correlation with the advancements in RT technology. From traditional 3-D conformal RT, lung cancer treatment methodologies have transitioned to sophisticated techniques like static IMRT. Asakura et al. (2) had reported that a larger volume of lung receives lower doses because of multiple beam arrangement and a smaller volume of lung receives higher doses because of better dose conformity in IMRT plans. Acute pneumonitis correlates more with V30 values, whereas chronic pneumonitis was predominantly seen in patients with higher V20 values. Recent innovations, such as VMAT (exemplified by Rapid Arc) and spiral Tomotherapy (TOMO), renowned for rapid treatment delivery, have broadened the low-dose irradiation expanse within the lung. Yaqin Zhao et al. showed that compared with IMRT, VMAT could increase the risk of acute radiation pneumonitis (72.73% vs. 36.96%) (P< 0.05) (6), Zhao Y reported that lung infection during radiotherapy, use of VMAT, mean lung dose(MLD), and dosimetric parameters (e.g. V20, V30) are significantly correlated with RP (6). Similar work has been reported by ZHU Shuchai et al. that the low dose volume of V5 is effective in predicting ≥grade 2 acute RP in patients with middle and lower thoracic esophageal cancer (7). These developments have inadvertently heightened the radiation doses in the lung tissue surrounding tumors, leading to a non-uniform radiation distribution.

lung has a parallel tissue architecture consisting of several independently functioning sub-units (FSUs). Accurately deriving such a dose–effect relationship through NTCP modeling of RP incidence is statistically challenging due to the increased number of degrees-of-freedom. As it is not possible to measure the damage to every single FSU, surrogates such as perfusion loss, ventilation and tissue density changes are used in the literature to find the local dose–effect relation. Selvaraj, Jothybasu et al. (5) reached a conclusion that the EUD perfusion based NTCP model had the lowest Akaike information criteria while the highest Akaike weights compared with V-x, MLD, which suggests that equivalent uniform dose (EUD) has certain advantages over traditional physical dose-based indicators in predicting radiation pneumonitis. In addition, the biological dose background of EUD can more accurately reflect the comprehensive damage of lung functional subunits (FSU) under non-uniform dose distribution. The EUD concept appears as a robust radiobiological surrogate of the dose distribution to select the optimal patient’s plan (12).

Historically, RP 2+ prediction was anchored on conventional RT dosimetric factors. Cunliffe et al. (13) proposed EUD as an independent predictor for RP 2+, but its predictive strength fell short compared to traditional factors like V20. Asakura’s study primarily hinged on the EUD formula introduced by Collier DC et al. (14) which overlooked the critical biological characteristic parameter α, possibly explaining the observed predictive variance.

A prospective, multi-institutional research focused on the dependence of radiation pneumonitis (RP) on generalized equivalent uniform dose (gEUD) was carried by E. L. Williams et al. (15) The gEUD was calculated for values of the volume parameter (a) ranging from log10a=-1.0 to +1.0 in steps of 0.1. The Lyman-Kutcher-Burman (LKB) model was fit to the RP data as a function of a. We determined the range of a where gEUD was significantly correlated with RP, the likelihood profile for the model fits and the best fit parameters. They found that the best fit α was 0.63 [0.32 - 1.02]. While, our study underscores a pronounced difference in relative lung EUD values between groups when α is set at 0.3 which very closed to the lower limit of the 95% confidence interval. This result was also verified by another research carried by Fan Liu et al. (15) which found the best fit values of the volume effect parameter α with upper 95% confidence limit around 1.0 in the joint data. While acknowledging potential calculation errors, it’s evident that the optimal α value for lung radiotherapy lung EUD lies between 0 and 1. This range holds clinical and research significance. Although literature often suggests an α value of 1 (16, 17). for parallel organs, the right selection remains crucial in radiotherapy contexts.

To the best of our knowledge, many studies have not clarified the rationale behind setting the α-value to 0.99 or 1 (12),despite the closely aligned calculated lung EUD and lung mean dose MLD. Jianrong Dai et al. (18) reported in their work that the equivalent uniform dose (EUD) derived from parallel/serial NTCP of the contralateral lung was identified as the second significant factor for RP2+ with an AUC of 0.744. In their study, the α-value was set to 0.99, which was close to the results of MLD (α-value equals to 1). However, our research revealed that the EUD value is highly sensitive to the α-value.

T.Hinton also proposed a method by combing miRNA and cytokine data along with generalized equivalent uniform dose (gEUD) to identify pathways with better accuracy of predicting RP2+ as compared to either miRNA or cytokines alone (19). From their research it was found that the prediction performance of RP 2+was significantly improved when the lung_gEUD was adopted both in pre-treatment and during treatment (4 weeks after treatment). However, the article did not give the specific calculation method of gEUD and clarified the α-value which will greatly affect the calculation results of gEUD, thus affecting the model prediction results.

Asakura et al (2). postulated that the number of radiation fields in thoracic tumor radiotherapy could predict RP 2+. Given that our study’s participants underwent VMAT, we analogized the arc range of VMAT to traditional IMRT’s radiation fields. However, our analysis found no significant difference in total irradiation arc or SAA between the groups. Extensive meta-analysis has highlighted distinct average PTVs between groups with and without RP 2+. However, it’s essential to discern the ratio of PTV volume to total lung tissue, which denotes how extensively radiotherapy affects the lung. Our study incorporated this ratio in regression analysis, but results differed from those of De Petris et al. (20) possibly due to our study’s limited sample size.

Perfect independence among variables is rarely achievable in real-world scenarios. Some variables might show significance in univariate analysis but lose this significance in multivariate contexts. This could be attributed to these variables being overshadowed by other variables in regression models. In our study, even with a significant correlation between lung EUD and physical dose, both metrics maintained their distinct roles in regression analysis. Hence, using both in clinical contexts can potentially enhance RP 2+ predictive accuracy.

This study’s simulation calculations inherently introduced errors in the lung EUD data. Additionally, patient positioning inaccuracies during treatment might have influenced data accuracy. Addressing these errors requires further data acquisition and detailed analysis.

Four prominent machine learning algorithms were used to devise our prediction models. The inclusion of lung EUD-based factors substantially enhanced their predictive performance for RP 2+. Our results advocate for the decision tree model with lung EUD-based predictors as the optimal prediction tool for VMAT-treated lung cancer patients. Emerging trends focus on combining lung dosimetric parameters with CT image-based radiomics as well as TGF-beta1 to predict radiation-induced side effects has widely reported (21). Our findings suggest lung EUD could replace conventional dosimetric parameters, potentially simplifying complex neural network structures in prediction models.

## 5 Conclusion

This study identified an optimal lung EUD characteristic parameter of 0.3, which accentuated the relative difference in lung EUD values between groups with and without RP 2+. Compared to conventional metrics, lung EUD (set at 0.3) showcased heightened clinical predictive capacities for RP 2+ under non-uniform irradiation. Furthermore, lung EUD-calibrated NTCP displayed superior predictive potential over the LKB model-based NTCP. The decision tree model, enriched with lung EUD predictors, demonstrated outstanding predictive capabilities, advocating its application in forecasting RP 2+ in lung cancer patients undergoing VMAT.

## Data availability statement

The raw data supporting the conclusions of this article will be made available by the authors, without undue reservation.

## Ethics statement

The study was conducted in accordance with the Declaration of Helsinki and approved by the Ethics Committee of Hangzhou Tumor Hospital (protocol code HCH/IEC AF/SG-04/03.0 and date of approval 17 October 2022). Informed consent was obtained from all subjects involved in the study.

## Author contributions

FY: Conceptualization, Methodology, Visualization, Writing – original draft. LX: Formal analysis, Investigation, Methodology, Writing – review & editing. YR: Data curation, Formal analysis, Methodology, Project administration, Software, Writing – review & editing. BX: Data curation, Methodology, Project administration, Validation, Writing – review & editing. XC: Formal analysis, Project administration, Writing – review & editing. SM: Formal analysis, Validation, Writing – review & editing. QD: Formal analysis, Resources, Conceptualization, Writing – review & editing. XL: Conceptualization, Data curation, Formal analysis, Funding acquisition, Supervision, Validation, Writing – original draft, Writing – review & editing.
